# Clinical and morphological features of SARS-COV-2 associated acute hemorrhagic necrotizing encephalopathy: case report

**DOI:** 10.1186/s41983-021-00413-1

**Published:** 2021-11-27

**Authors:** Victor Vladimirovich Ermilov, Nikita Alexeevich Dorofeev

**Affiliations:** 1 Department of Forensic Medicine, Volgograd State Medical University, Volgograd, Russia 400131; 2 Laboratory of Informatization and Digitalization of Healthcare, Volgograd Medical Research Center, Volgograd, Russia 400131

**Keywords:** SARS-CoV-2, Acute hemorrhagic necrotizing encephalopathy, Endothelial dysfunction, Endotheliitis

## Abstract

**Background:**

The current case report presents acute hemorrhagic necrotizing encephalopathy (AHNE) as an example of a fatal complication, the etiology of which could be coronavirus disease 2019 (COVID-19) with multiple organ damage along with the existing respiratory tuberculosis.

**Case presentation:**

A male in his 20s had severe symptoms of central nervous system lesion, which developed against the background of COVID-19 and respiratory tuberculosis, for which he was treated in the intensive care unit. Autopsy confirmed that he died from severe acute respiratory syndrome coronavirus 2 (SARS-CoV-2) associated AHNE in adults with severe fatal endothelial dysfunction and respiratory tuberculosis. The main morphological signs of brain damage were desquamative endotheliitis, thrombosis, parenchymal hemorrhagic necrosis, encephalitis, severe necrobiotic neuronal damage.

**Conclusion:**

The defeat of endothelial cells with the development of generalized endotheliitis in COVID-19, especially in conjunction with comorbid pathology, in particular tuberculosis, can lead to a fatal complication that affects the nervous system—AHNE. Therefore, it is worth paying close attention to the appearance of neurological symptoms in patients with a similar combination of diseases.

## Background

COVID-19 is an acute infectious disease caused by SARS-CoV-2, which can lead to the development of severe acute respiratory syndrome [1, 2]. Scientific literature already contains references to multiple organ lesions associated with COVID-19, including those affecting nervous system [1, 2]. This case report presents AHNE as an example of a fatal complication that could be etiologically related to a novel coronavirus infection as well as a patient’s comorbid pathology.

## Case presentation

The patient was born in a blood marriage. About 3 years ago, he had hand tremors and headache, which indicates the possibility of a previously existing lesion of the nervous system. The current episode of disease began, when the patient in his 20s had a fever. He called an ambulance after learning about it, but refused hospitalization. The next day, when a rise in body temperature to 38 °C was noted, he was hospitalized in a primary hospital, where a positive test for COVID-19 was obtained by polymerase chain reaction (PCR). Then he was hospitalized in an infectious diseases hospital (IDH), where he stayed for 15 days with diagnosis: “COVID-19 of moderate severity. Left-sided pneumothorax. Focal symptomatic epilepsy”.

Chest computed tomography performed at the IDH showed infiltrative pulmonary tuberculosis in the phase of disintegration and seeding, left-sided pneumothorax.

C-reactive protein level was steadily increased—from 54 to 220 mg/l (range 0–5). Cerebrospinal fluid protein level was elevated on the first measurement—0.9 g/l (range 0.22–0.33), but returned to normal when repeated after 8 days.

**Fig. 1:**
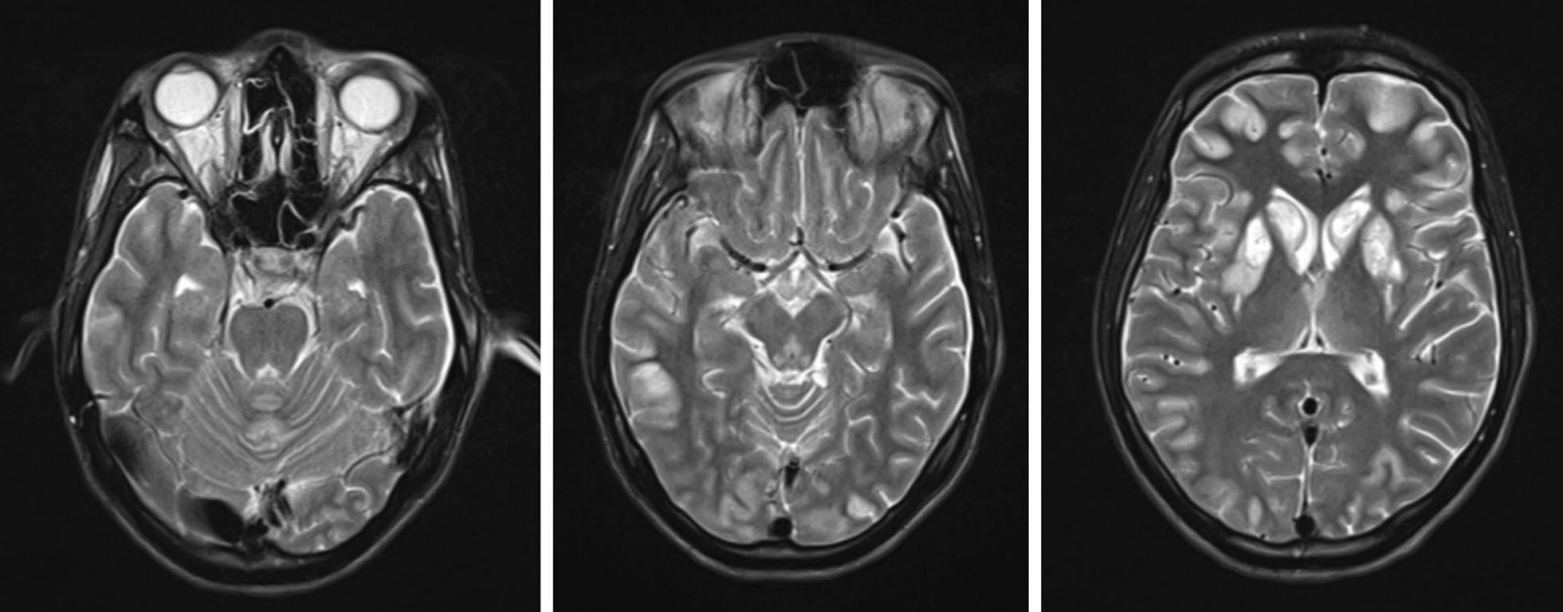
MRI of the brain. Narrowing of the subarachnoid space. A pathological increase in the intensity of the signal by T2/TIRM and DWI from the cortex of the brain substance is determined, the change in the signal is symmetrical from the basal nuclei with their hemorrhagic transformation. Symmetrically increased T2/TIRM signal from the medial thalamus, periaqueductal gray matter

On the 15th day of treatment in an IDH, he was taken to the intensive care unit of an antituberculosis dispensary in an extremely serious condition. During his stay at the dispensary, magnetic resonance imaging (MRI) scan of the brain was performed. In the conclusion of MRI of the brain described the MRI picture of acute necrotizing encephalopathy (Fig. 1). After that the neurologist diagnosed “Acute necrotizing encephalopathy of adults with mixed infection, impaired consciousness and focal epileptic syndrome” and noted weakly positive meningeal symptoms.

Among laboratory tests were a positive sputum analysis for *Mycobacterium tuberculosis* by PCR and fluorescence microscopy, semi-quantitative procalcitonin 2–10 ng/ml (negative < 0.064), analysis for IL-6—420.4 pg/ml (negative < 7), as well as lymphopenia in the absence of leukocytosis. An immunological examination was carried out. According to its results, significant lymphopenia was revealed (on average, ten times less than the norm in each type of lymphocytes).

After long-term treatment with negative dynamics, on the 7th day of stay in the intensive care unit, the patient died.

Autopsy during examination of the cranial cavity revealed cerebral edema, focal hemorrhages and areas of disturbance of the brain structure in the bottom of the interhemispheric groove, anterior poles of the frontal lobes and at the base of the temporal lobes, as well as in the cortex, white matter and basal nuclei.

**Fig. 2:**
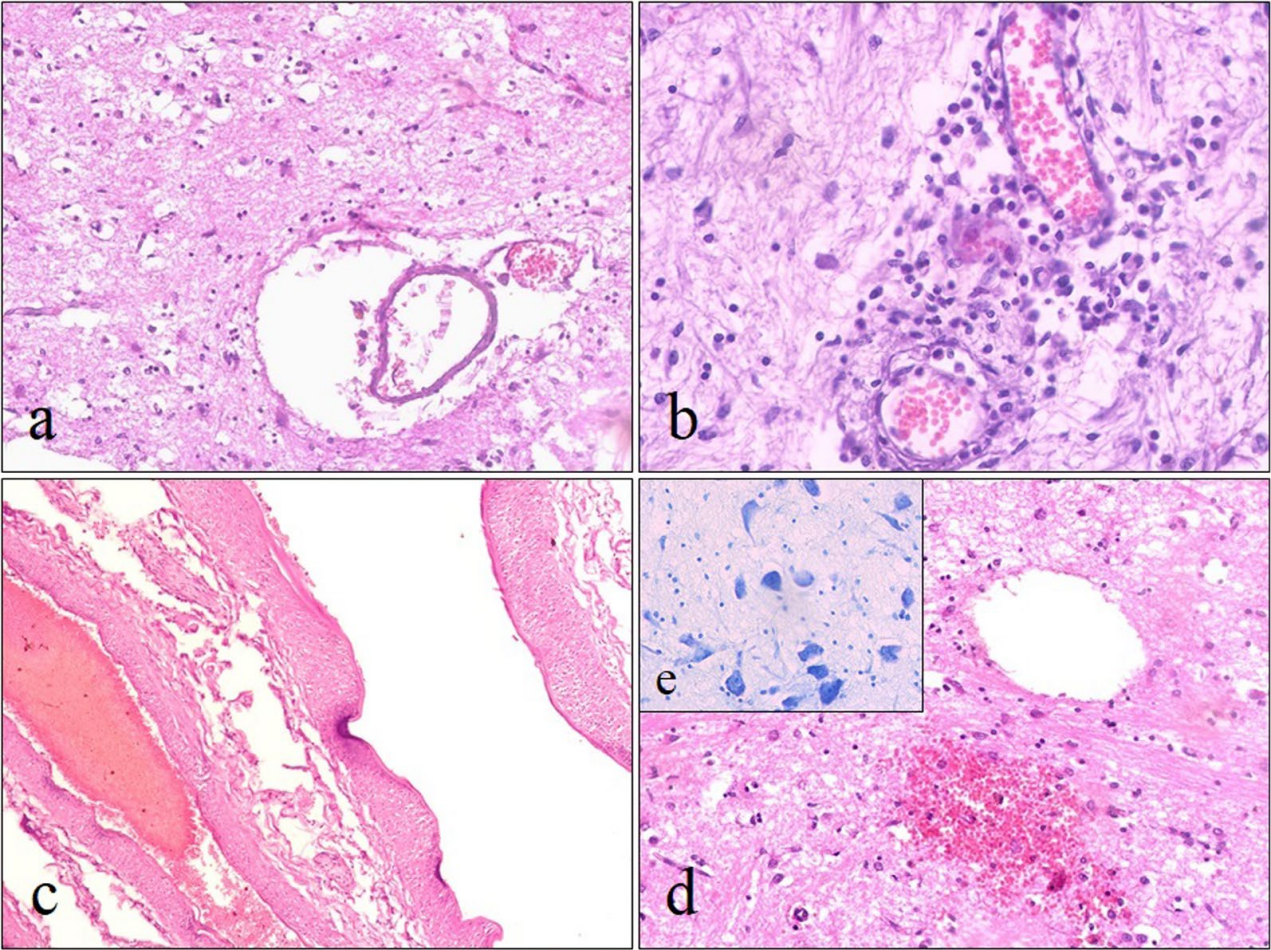
Histological picture of brain lesions. **a**, **b** Inflammatory infiltration, spongy and perivascular edema, areas of necrosis, desquamation of the endothelium in the cerebral vessels. ×200. **c** Fibrinoid necrosis of cerebral vessels. H&E staining. ×100. **d** AHNE with the formation of a cyst. H&E staining. ×200. **e** Severe necrobiotic neuronal damage. Nissl staining. ×400

Histological examination of the brain revealed morphological signs of widespread vasculitis with inflammatory cellular infiltration of the vessel walls by mononuclear cells, lymphocytes and neutrophils, with the presence of perivascular cuffs and edema, with the spread of cellular infiltrate into the gray and white matter of the brain (Fig. 2a, b).

The pathological process mainly affected small vessels with varying degrees of segmental and total destruction of the endothelium, its swelling, desquamation, fibrinoid necrosis of the walls and pronounced perivascular edema (Fig. 2a–c). Sludge, thrombosis with fibrinous, erythrocytic and mixed thrombi with concomitant signs of inflammation or without them, with perivascular hemorrhages were also noted in these vessels.

In the gray and white matter areas of parenchymal, mainly hemorrhagic, necrosis with pronounced perifocal spongy edema with the formation of microcysts, small focal hemorrhages and glial nodules as a reaction to parenchymal inflammation and severe necrobiotic neuronal damage were determined (Fig. 2b, d, e).

Histological examination of other organs also revealed multiple comorbid pathology, represented by infiltrative pulmonary tuberculosis, tuberculous pleurisy, acute bilateral focal-confluent bronchopneumonia, erosive–desquamative tracheobronchitis, acute respiratory distress syndrome in adults, disseminated intravascular coagulation, interstitial myocardial edema, necrotic nephrosis, centrilobular necrosis in the liver, pancreatic desquamative endotheliitis, lymphoid depletion of the spleen.

## Discussion

Neurological disorders, according to the scientific literature, can be associated with COVID-19, determining the severity of the disease in some cases [1, 2]. The presented case is an example of the simultaneous effect of two pathogens on the human body, which, even separately, can lead to death. Given the limited possibilities of extended laboratory and instrumental diagnostics, as well as immunohistochemical examination, it is impossible to say unequivocally what role SARS-CoV-2 played in the development of AHNE in this case. However, the results of the histological examination showed that the microscopic picture corresponded to the positive dynamics of the course of tuberculosis during treatment. Summarizing all these facts, the authors suggest that the death of the patient, as well as the severity of the disease, was largely due to COVID-19.

## Conclusion

The defeat of endothelial cells with the development of generalized endotheliitis in COVID-19, especially in conjunction with comorbid pathology, in particular tuberculosis, can lead to a fatal complication that affects the nervous system—AHNE. Therefore, it is worth paying close attention to the appearance of neurological symptoms in patients with a similar combination of diseases.

## Data Availability

Not applicable.

## Abbreviations

AHNE: Acute hemorrhagic necrotizing encephalopathy
COVID-19: Coronavirus disease 2019
SARS-CoV-2: Severe acute respiratory syndrome coronavirus 2
PCR: Polymerase chain reaction
IDH: Infectious diseases hospital
MRI: Magnetic resonance imaging
H&E: Hematoxylin and eosin
